# The relationship between personality traits and happiness: the mediating role of emotional regulation

**DOI:** 10.1186/s12912-024-01959-0

**Published:** 2024-05-14

**Authors:** Emad Shdaifat, Tamadur Shudayfat, Amira Alshowkan

**Affiliations:** 1 Community Nursing Department, College of Nursing, Imam Abdulrahman Bin Faisal University,, P.O Box 1982, Dammam, Saudi Arabia; 2 Community and Mental Health Nursing Department, Faculty of Nursing, Al al-Bayt University, P. O. Box 130040, Mafraq, Jordan

**Keywords:** Personality traits, Emotional regulation, Happiness, Cultural differences, Jordan, Saudi Arabia

## Abstract

**Background:**

Understanding the factors contributing to happiness in the nursing profession is essential, particularly considering the high levels of stress associated with the job. This study aimed to explore the role of emotion regulation in mediating the relationship between personality traits and nurses’ happiness.

**Objective:**

This study aimed to explore the relationship between personality traits and happiness by examining the mediating role of emotion regulation.

**Methods:**

A cross-sectional study was conducted with 324 Jordanian and 408 Saudi nurses. Data, including the Big Five personality traits, happiness levels, and measures of emotional regulation, were collected through an online survey. The model’s fit and explanatory capability were verified by Structural Equation Modeling (SEM) using SmartPLS 3.

**Results:**

In the structural model, agreeableness had a significant effect on happiness, influencing both reappraisal and suppression. Extraversion strongly influences happiness, positively affects reappraisal, and negatively affects suppression. Neuroticism hampers happiness and reappraisal, and has a detrimental effect on suppression. Openness had a positive effect on suppression, whereas consciousness positively affected happiness. Mediation analysis revealed direct effects on happiness, with varying indirect contributions from emotional regulation. Multiple-group analysis revealed no significant differences between Jordan and Saudi Arabia in the association between personality traits and happiness.

**Conclusion:**

The findings emphasize the nuanced effects of agreeableness, extraversion, neuroticism, consciousness, and openness on happiness, mediated by emotional regulation. Implementing specific interventions to improve emotional regulation can increase nurses’ happiness regardless of their personality traits. The lack of significant differences between Jordanian and Saudi nurses implies that these relationships are consistent across cultures, offering valuable information for cross-cultural healthcare interventions.

## Introduction

Happiness is a crucial aspect of overall well-being, affecting different aspects of life such as performance, health, and social interactions [1–3]. Rooted in positive psychology theories, happiness is defined as feelings of joy, prosperity, and contentment, which contribute to personal growth and positive social relationships [4, 5]. De-Juanas et al. (2020) describe happiness as a multidimensional concept encompassing autonomy, purpose, and personal growth. Autonomy, the ability to make independent choices, is closely tied to psychological well-being [6].

The concept of happiness has expanded beyond simply acquiring material possessions. It now incorporates the principles of the self-determination theory, which emphasizes intrinsic goals and the experience of positive emotions [7]. Cultural influences on happiness and goal pursuit are significant, with each culture shaping its own unique definition and expression of happiness [8]. Research has consistently linked happiness to better earnings, health, and marital status [9–11].

Personality traits, particularly the Big Five, significantly influence happiness [12] Traits like emotional stability, conscientiousness, extraversion, and agreeableness correlate with higher life and work satisfaction, strengthening with age. Based on the process model, emotional regulation is seen as a key factor in determining happiness and overall well-being [13, 14]. This perspective emphasizes aligning experienced emotions with desired emotions through conscientious efforts [15].

Nurses working in emotionally demanding environments can greatly benefit from gaining a thorough understanding of their personality traits and emotional regulation strategies, as highlighted by extensive research [16]. Investigating these connections within different cultural settings, as recommended by previous studies, offers the invaluable insights necessary for the development of effective cross-cultural healthcare interventions.

In summary, the complex relationship among personality traits, emotional regulation, and happiness underscores the importance of understanding personal traits to support well-being. By investigating the mediating role of emotional regulation, this study aimed to explore this relationship among nurses in Jordan and Saudi Arabia. Through the lens of emotion regulation, we hope to shed light on how personality traits affect happiness and make a significant contribution to the fields of psychology and healthcare research. Comprehending these relationships is essential, especially in professional environments in which individuals navigate intricate emotional terrains. This research aimed to clarify the processes underlying happiness and emotional regulation (reappraisal and suppression) and offer practical recommendations for improving emotional health in various settings.

**Fig. 1:**
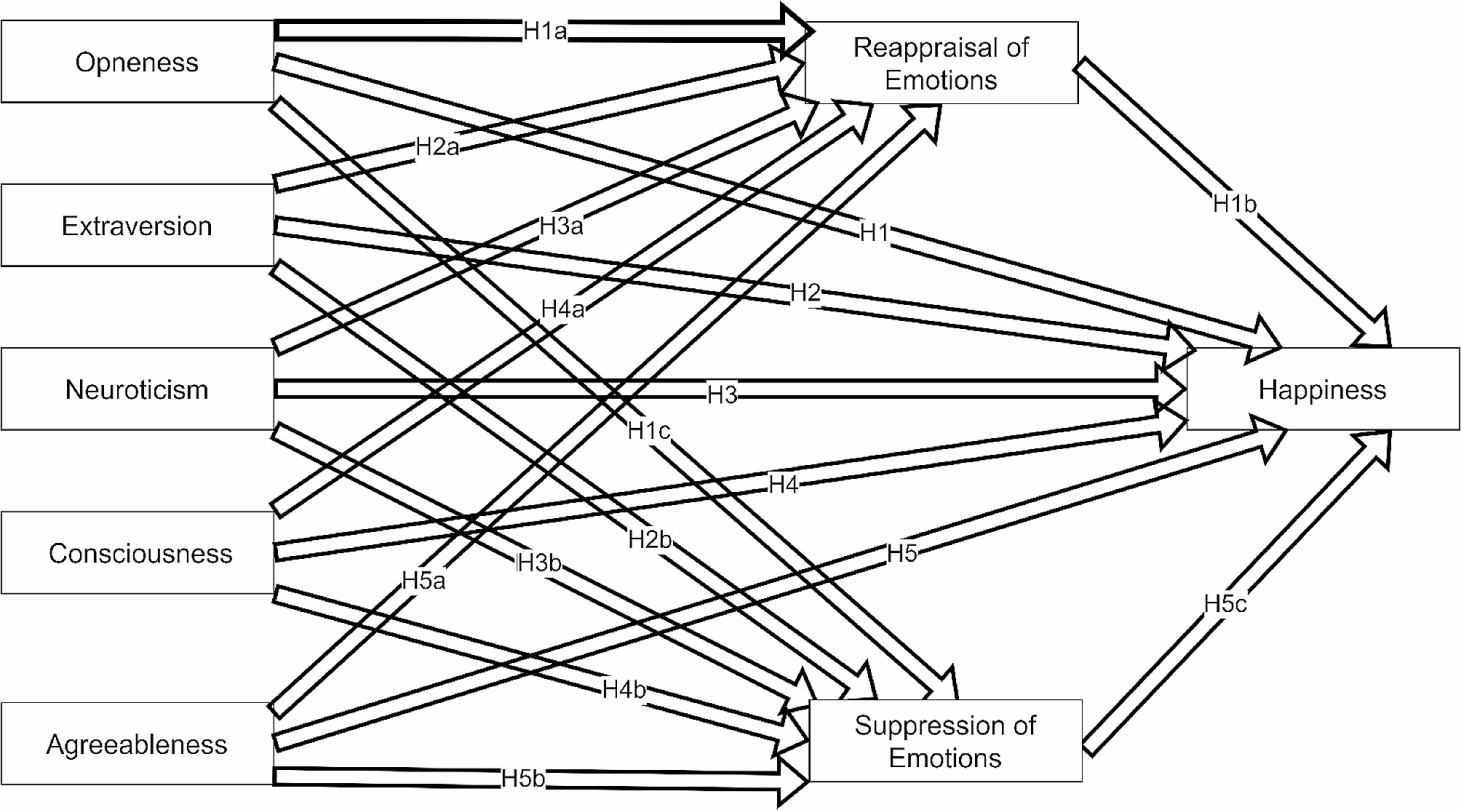
Conceptual model

## Hypothesis

### H1

 Openness has a significant effect on happiness.

### H1a

 Openness has a significant effect on reappraisal.

### H1b

 Reappraisal has a significant effect on happiness.

### H1c

 Openness has a significant effect on suppression.

### H2

 Extraversion had a significant effect on happiness.

### H2a

 Extraversion had a significant effect on reappraisal.

### H2b

 Extraversion had a significant effect on suppression.

### H3

 Neuroticism has a significant effect on happiness.

### H3a

 Neuroticism has a significant effect on reappraisal.

### H3b

 Neuroticism has a significant effect on suppression.

### H4

 Consciousness has a significant effect on happiness.

### H4a

 Consciousness has a significant effect on reappraisal.

### H4b

 Consciousness had a significant effect on suppression.

### H5

 Agreeableness has a significant effect on happiness.

### H5a

 Agreeableness has a significant effect on reappraisal.

### H5b

 Agreeableness had a significant effect on suppression.

### H5c

 Suppression has a significant effect on happiness.

Personality traits have direct and indirect impacts on happiness through emotional regulation. This study investigated how emotional regulation acts as a mediator between personality traits and happiness. Hypotheses were formulated to reflect these dynamics. Accordingly, the hypotheses were formulated (Fig. 1).

## Methods

### Setting

This cross-sectional study was conducted at the Nursing College of Al al-Bayt University in northern Jordan and Imam Abdulrahman Bin Faisal University in eastern Saudi Arabia. Data were collected from March to September 2023. This study was conducted as part of a research project to compare the levels of happiness among nurses in Saudi Arabia and Jordan.

### Participants

This study recruited nursing students from our college from the first to the fifth year. To determine the sample size, the G* Power software was used. With an effect size (d) of 0.2, significance level (α) of 0.05, and desired power (1-β) of 0.80, each group required a sample size of 310 based on an allocation ratio of 1. Consequently, the total sample size of this study was 620.

### Data collection tools

Three instruments were used in the study. First, the Big Five Personality Questionnaire, which employs the 44-item short version of the Big Five Inventory, uses a Likert scale ranging from 1 (strongly disagree) to 5 (strongly agree) to evaluate subjects’ personalities across five subscales: Consciousness, Extraversion, Agreeableness, Openness, and Neuroticism [17]. The validity and reliability of the Arabic version were established, with Cronbach’s alpha ranging from 0.84 to 0.68 for the subscales [18], and reverse coding was applied to negative items. Second, the Oxford Happiness Inventory (OHI), a 29-item tool with responses ranging from 1 (strongly disagree) to 6 (strongly agree), was used to assess the levels of happiness [19]. The validity and reliability of the Arabic version were confirmed, with Cronbach’s alpha ranging from 0.91 to 0.89 for the subscales [18]. The scores were totalled and divided by 29 after reverse coding of certain items. Finally, the Emotional Regulation Questionnaire (ERQ): Emotional regulation was measured using the 10-item ERQ, which includes two subscales, reappraisal and suppression, with Likert scale ratings ranging from 1 (strongly disagree) to 5 (strongly agree) [20].

### Consideration of ethical principles

The Institutional Review Board (IRB) at Al al-Bayt University and Imam Abdulrahman Bin Faisal University provided ethical approval for this study. Approval for the study methodology and surveys was obtained before recruitment. In addition to verbal communication, the students were provided with a detailed information sheet that clarified the voluntary nature of their participation, ensured their right to withdraw at any time without explanation, and guaranteed that their rights and learning would not be affected. The document also outlines the study’s significance, benefits, and objectives.

### Data analysis

This study utilized SmartPLS 3 for data analysis, including the assessment of factor loadings, composite reliability, and extraction of mean variance for convergent validity. Before conducting structural equation modeling (SEM), The assumptions of normality and multicollinearity were verified before conducting SEM. SEM analysis was carried out using SmartPLS 3.0, exploring the relationships among latent constructs: personality traits, emotional regulation, and happiness, considering both direct and indirect effects. Bootstrapping, with a significance level of *p* < 0.05, and a minimum of 5,000 resamples, was used to determine the confidence intervals and significance. The SEM results were used to evaluate the hypothetical model and investigate the potential mediating role of emotional regulation in the relationship between personality traits and happiness. Moreover, this study employed Henseler’s Multigroup Analysis (MGA) to compare the correlation between personality traits and happiness among nurses in Jordan and Saudi Arabia. MGA, a non-parametric test, evaluates group-specific variances using PLS-SEM bootstrapping. Significance is established at a 5% error probability, where a *p*-value below 0.05 or above 0.95 indicates a noteworthy disparity in group-specific path coefficients. The PLS-MGA methodology in SmartPLS expands on bootstrap-based MGA for PLS-SEM, offering a cautious assessment of the path coefficient differences between the two groups [21, 22].

## Results

### Characteristics of participants

Most participants in this study were from Saudi Arabia, comprising 55.9% of the total sample. In terms of sex distribution, males constituted 47.0%, while individuals aged 20 years or older constituted 51.2%. Regarding marital status, the majority of the participants (95.8%) were single. Regarding education, participants were distributed across various academic years, with 44.4% in their first year.

### Measurement model

Construct reliability was assessed using Cronbach’s alpha and composite reliability (CR), with all CR values exceeding the recommended threshold of 0.70 [23]. Cronbach’s alpha for each construct surpassed the threshold of 0.63 for each construct. Convergent validity, evaluated using Average Variance Extracted (AVE), ranged from 0.50 to 0.69. According to Fornell and Larcker (1981), these values indicate a satisfactory convergent validity [24]. The factor loadings, as presented below, were consistent with the results of Hair et al. (2006), where the minimum interpretability level for the structure ranges from 0.30 to 0.40 [25]. All factors and their respective subscales exhibited a high level of significance. Table 1 provides a comprehensive overview of the reliability and validity results, as well as the factor loadings of the items. Additionally, the variance inflation factor (VIF) ranged between 1.13 and 2.06 (Table 1).

**Table 1:** Reliability and validity analysis

Construct	Item	Loading	Cronbach’s Alpha	rho_A	Composite Reliability	AVE	VIF
Happiness	Happ15	0.800	0.800	0.800	0.860	0.500	1.21
	Happ22	0.720					1.57
	Happ7	0.720					1.56
	Happ12	0.700					1.49
	Happ9	0.690					1.54
	Happ18	0.610					2.06
Suppression	Support9	0.880	0.780	0.830	0.870	0.690	1.55
	Support8	0.840					1.85
	Support7	0.780					1.60
Reappraisal	Reapp2	0.810	0.780	0.790	0.850	0.530	1.39
	Reapp3	0.800					1.28
	Reapp1	0.730					1.38
	Reapp6	0.650					1.97
	Reapp4	0.640					1.94
Agreeableness	Agree32	0.740	0.670	0.670	0.800	0.500	1.25
	Agree42	0.710					1.26
	Agree17	0.690					1.21
	Agree7	0.690					1.32
Consciousness	Cons28	0.790	0.710	0.730	0.820	0.540	1.20
	Cons33	0.780					1.41
	Cons3	0.690					1.51
	Cons38	0.650					1.39
Extraversion	Extrav11	0.860	0.630	0.700	0.800	0.570	1.13
	Extrav16	0.810					1.40
	Extrav36	0.570					1.36
Neuroticism	Nuro24	0.800	0.760	0.780	0.830	0.510	1.39
	Nuro34	0.750					1.48
	Nuro39	0.720					1.53
	Nuro9	0.710					1.41
	Nuro19	0.550					1.68
Openness	Open5	0.830	0.700	0.730	0.810	0.530	1.18
	Open15	0.740					1.37
	Open25	0.720					1.33
	Open20	0.600					1.54

The Fornell-Larcker criterion evaluates discriminant validity. The diagonal values (bold) indicate the square roots of AVE for each construct. Discriminant validity is deemed satisfactory when these values exceed the off-diagonal correlations. In Table 2, discriminant validity seems to be supported because the bold diagonal values are higher than their corresponding off-diagonal correlations.

**Table 2:** Fornell-larcker criterion discriminant validity

	Agreeableness	Reappraisal	Consciousness	Extraversion	Happiness	Neuroticism	Openness	Suppression
Agreeableness	***0.710***							
Reappraisal	0.390	***0.730***						
Consciousness	0.450	0.320	***0.730***					
Extraversion	0.470	0.380	0.460	***0.760***				
Happiness	0.380	0.360	0.380	0.570	***0.710***			
Neuroticism	-0.180	-0.370	-0.250	-0.280	-0.400	***0.710***		
Openness	0.360	0.300	0.540	0.530	0.360	-0.230	***0.730***	
Suppression	0.210	0.280	0.190	0.060	0.100	-0.340	0.170	***0.830***

*The bold italicized text is the square root of AVE*

The Heterotrait-Monotrait Ratio of Correlations (HTMT) was used to evaluate discriminant validity. Values exceeding one indicate potential issues with discriminant validity. In the table, all HTMT values are below 1, which indicates satisfactory discriminant validity. These constructs exhibited an appropriate level of differentiation, thereby supporting the distinctiveness of the measured traits (Table 3).

**Table 3:** Heterotrait-monotrait ratio of correlations (HTMT) discrimination validity of the measurement model

	Agreeableness	Reappraisal	Consciousness	Extraversion	Happiness	Neuroticism	Openness
Reappraisal	0.540						
Consciousness	0.640	0.410					
Extraversion	0.720	0.510	0.660				
Happiness	0.510	0.450	0.490	0.750			
Neuroticism	0.260	0.430	0.310	0.380	0.520		
Openness	0.520	0.390	0.740	0.770	0.460	0.340	
Suppression	0.290	0.350	0.250	0.090	0.110	0.380	0.230

The model fit statistics in Table 4 provide insight into the adequacy of the proposed model. A Standardized Root Mean Square Residual (SRMR) of 0.070 suggests a good fit. The Unweighted Least Squares discrepancy (d_ULS) and Bentler’s Comparative Fit Index (d_G) values at 3.030 and 0.590, respectively, indicate an acceptable fit. However, the high chi-square value of 2507.470 raises concerns and suggests a potential lack-of-fit. It is important to interpret this cautiously, in conjunction with other fit indices. Notably, the Normed Fit Index (NFI) of 0.700 falls below the commonly accepted threshold of 0.90, suggesting a possible need for model refinement. Further examination and potential adjustments of the model may enhance its overall fit and validity.

**Table 4:** Assessment of model fit statistics

	SRMR	d_ULS	d_G	Chi-Square	NFI
Saturated Model	0.070	2.960	0.590	2495.220	0.700
Estimated Model	0.070	3.030	0.590	2507.470	0.700

Table 5 presents the values of R-square (R²) and Q Square (Q²). In terms of happiness, 41% of the variance was explained (R² = 0.41) and it had a predictive relevance of 20% (Q² = 0.20). Reappraisal had an explained variance of 27% (R² = 0.27) and a predictive relevance of 14% (Q² = 0.14). For Suppression, the explained variance was 17% (R² = 0.17) and the predictive relevance was 10% (Q² = 0.10). These values demonstrate the model’s ability to explain and predict variance in the dependent variables.

**Table 5:** The value of R-square and Q square

	*R* ^2^	Q²
Happiness	0.41	0.20
Reappraisal	0.27	0.14
Suppression	0.17	0.10

### Structure model

Table 6 presents the essential path coefficients and results of the hypothesis testing in the structural equation model. Notably, Agreeableness significantly enhanced happiness (β = 0.10, *p* < 0.001), demonstrating substantial positive effects on reappraisal (β = 0.24, *p* < 0.001) and suppression (β = 0.18, *p* < 0.001). Extraversion strongly influenced happiness (β = 0.39, *p* < 0.001), with a positive impact on reappraisal (β = 0.14, *p* < 0.001) and a negative effect on suppression (β = -0.21, *p* < 0.001). Neuroticism significantly impeded happiness (β = -0.25, *p* < 0.001) and reappraisal (β = -0.26, *p* < 0.001), while having a detrimental effect on suppression (β = -0.33, *p* < 0.001). Openness had a noteworthy positive effect on suppression (β = 0.11, *p* = 0.01). Consciousness demonstrated a modest, yet significant, positive impact on happiness (β = 0.08, *p* = 0.04). However, its influence on reappraisal (β = 0.05, *p* = 0.23) and suppression (β = 0.06, *p* = 0.22) was not statistically significant. Notably, there was a negative correlation between extraversion and suppression (β = -0.21, *p* < 0.001), indicating that nurses who were more extraverted tended to engage in less emotional suppression. Likewise, neuroticism negatively affected both reappraisal (β = -0.26, *p* < 0.001) and suppression (β = -0.33, *p* < 0.001), suggesting that neurotic nurses are less likely to utilize these coping strategies. This overview outlines the relationships between personality traits - such as agreeableness, extraversion, neuroticism, consciousness and openness- and their contributions to happiness and emotional regulation in the model.

**Table 6:** The path coefficient and hypothesis testing

		Β	SD	T Statistics	*P* Values
H1	Openness -> Happiness	0.01	0.04	0.32	0.75
H1a	Openness -> Reappraisal	0.05	0.04	1.11	0.27
H1b	Reappraisal -> Happiness	0.08	0.04	2.14	0.03
H1c	Openness -> Suppression	0.11	0.05	2.5	0.01
H2	Extraversion -> Happiness	0.39	0.04	8.94	< 0.001
H2a	Extraversion -> Reappraisal	0.14	0.04	3.43	< 0.001
H2b	Extraversion -> Suppression	-0.21	0.05	4.3	< 0.001
H3	Neuroticism -> Happiness	-0.25	0.04	6.5	< 0.001
H3a	Neuroticism -> Reappraisal	-0.26	0.04	6.71	< 0.001
H3b	Neuroticism -> Suppression	-0.33	0.04	8.44	< 0.001
H4	Consciousness -> Happiness	0.08	0.04	2.01	0.04
H4a	Consciousness -> Reappraisal	0.05	0.04	1.21	0.23
H4b	Consciousness -> Suppression	0.06	0.05	1.22	0.22
H5	Agreeableness -> Happiness	0.10	0.04	2.87	< 0.001
H5a	Agreeableness -> Reappraisal	0.24	0.04	5.9	< 0.001
H5b	Agreeableness -> Suppression	0.18	0.04	4.26	< 0.001
H5c	Suppression -> Happiness	-0.07	0.04	1.86	0.06

Table 7 presents a summary of the total effects observed in the model, offering valuable insights into the strength and significance of these relationships. These findings highlighted several noteworthy results. First, agreeableness exhibited significant positive effects on reappraisal (β = 0.240, *p* < 0.001), happiness (β = 0.110, *p* < 0.001), and suppression (β = 0.180, *p* < 0.001). Second, extraversion demonstrated substantial positive effects on reappraisal (β = 0.140, *p* < 0.001) and happiness (β = 0.410, *p* < 0.001) while exhibiting a negative impact on suppression (β = -0.210, *p* < 0.001). Third, neuroticism had negative effects on reappraisal (β = -0.260, *p* < 0.001), happiness (β = -0.250, *p* < 0.001), and suppression (β = -0.330, *p* < 0.001). Finally, openness had a significant positive effect on suppression (β = 0.110, *p* = 0.010). These outcomes enhance our understanding of the direction and strength of the relationships within the model.

**Table 7:** Summary of total effects in the model

	β	SD	T Statistics	*P* Values
Openness -> Happiness	0.01	0.04	0.23	0.82
Openness -> Reappraisal	0.05	0.04	1.11	0.27
Reappraisal -> Happiness	0.08	0.04	2.14	0.03
Openness -> Suppression	0.11	0.05	2.5	0.01
Extraversion -> Happiness	0.41	0.04	9.73	< 0.001
Extraversion -> Reappraisal	0.14	0.04	3.43	< 0.001
Extraversion -> Suppression	-0.21	0.05	4.3	< 0.001
Neuroticism -> Happiness	-0.25	0.03	7.19	< 0.001
Neuroticism -> Reappraisal	-0.26	0.04	6.71	< 0.001
Neuroticism -> Suppression	-0.33	0.04	8.44	< 0.001
Consciousness -> Happiness	0.08	0.04	2.01	0.04
Consciousness -> Reappraisal	0.05	0.04	1.21	0.23
Consciousness -> Suppression	0.06	0.05	1.22	0.22
Agreeableness -> Happiness	0.11	0.03	3.15	< 0.001
Agreeableness -> Reappraisal	0.24	0.04	5.9	< 0.001
Agreeableness -> Suppression	0.18	0.04	4.26	< 0.001
Suppression -> Happiness	-0.07	0.04	1.86	0.06

### Mediation analysis

Table 8 presents the results of the mediation analysis, providing details of the direct, indirect, and total effects. In terms of agreeableness, there was a significant positive direct effect on happiness (β = 0.100, *p* < 0.001), but the indirect effect was not significant (β = 0.010, *p* = 0.590), resulting in a significant total effect of 0.110 (*p* < 0.001). Consciousness demonstrated a positive direct effect on happiness (β = 0.080, *p* = 0.040) with no significant indirect effect, leading to a significant total effect of 0.080 (*p* = 0.040). Extraversion displayed a substantial positive direct effect on happiness (β = 0.390, *p* < 0.001) with a small but significant indirect effect (β = 0.020, *p* = 0.020), resulting in a significant total effect of 0.410 (*p* < 0.001). Neuroticism exhibited a significant negative direct effect on happiness (β = -0.250, *p* < 0.001), with no significant indirect effect, leading to a significant total effect of -0.250 (*p* < 0.001). Openness demonstrated a nonsignificant direct effect on happiness (β = 0.010, *p* = 0.750) and no significant indirect effect, resulting in a nonsignificant total effect of 0.010 (*p* = 0.820). These findings offer valuable insights into the complex relationship between personality traits and happiness, presenting various patterns of direct and indirect effects.

**Table 8:** Path analysis: direct, indirect, and total effects

	Direct Effect		Indirect Effect		Total Effect	
	β	*P* Values	β	*P* Values	β	*P* Values
Agreeableness -> Happiness	0.100	< 0.001	0.010	0.590	0.110	< 0.001
Consciousness -> Happiness	0.080	0.040	0.000	0.980	0.080	0.040
Extraversion -> Happiness	0.390	< 0.001	0.020	0.020	0.410	< 0.001
Neuroticism -> Happiness	-0.250	< 0.001	0.000	0.870	-0.250	< 0.001
Openness -> Happiness	0.010	0.750	0.000	0.550	0.010	0.820

### The difference between Jordan and Saudi

Multiple-group analysis revealed no significant differences between Jordan and Saudi Arabia in the relationship between personality traits and happiness, reappraisal, and suppression. No difference in the path coefficients was statistically significant (*p* > 0.05), indicating similar patterns in these associations across the two countries (Table 9).

**Table 9:** Multiple-group analysis

	Path Coefficients-diff (Jordan - Saudi)	*P*-Value
Agreeableness -> Happiness	-0.040	0.583
Agreeableness -> Reappraisal	0.031	0.701
Agreeableness -> Suppression	0.010	0.910
Consciousness -> Happiness	-0.019	0.809
Consciousness -> Reappraisal	-0.078	0.397
Consciousness -> Suppression	0.027	0.781
Extraversion -> Happiness	-0.050	0.577
Extraversion -> Reappraisal	0.022	0.799
Extraversion -> Suppression	-0.088	0.360
Neuroticism -> Happiness	-0.066	0.395
Neuroticism -> Reappraisal	-0.062	0.430
Neuroticism -> Suppression	-0.101	0.194
Openness -> Happiness	0.107	0.194
Openness -> Reappraisal	0.031	0.726
Openness -> Suppression	0.031	0.731
Reappraisal -> Happiness	-0.063	0.396
Suppression -> Happiness	-0.088	0.235

## Discussion

This study aimed to explore the correlation between personality traits and happiness by specifically investigating the mediating role of emotional regulation in this relationship. This study found that agreeableness had a significant impact on happiness, influencing both reappraisal and suppression, which is supported by previous studies [26, 27]. This result can be justified as nursing students with agreeableness traits are considered more empathic and altruistic, and have a high intention to help others. These agreeable personality traits are the core values that nursing students learn during their first year of college and help enhance their self-esteem and nursing image. Therefore, agreeableness is thought to affect the levels of happiness and ability of nursing students to reappraise their emotions and suppression.

In addition, our study found a positive effect of agreeable personality and reappraisal, which is consistent with another study [28]. This suggests that agreeable people tend to use reappraisal to regulate the effects of antisocial stimuli Finlay-jones (2017), which is probably due to their engagement in reappraisal ability [29].

The current study revealed that extraversion strongly influences happiness and positively affects reappraisal. This result was supported by several studies [27, 30, 31]. The possible rationale for this finding is that extraversion personality traits increase interpersonal experience, enablement to enjoy, sharing feelings with others, and a preference for achievement rather than focusing on life experiences. Likewise, it was established that the maximum amount of time students spent participating in recreational activities and with their families was related to their levels of happiness [32]. Therefore, future research should explore the impact of family relationships and leisure activities on students’ happiness levels. In addition, as part of the nursing study curriculum, students are encouraged to participate in extracurricular activities, which may help them to be open and socially active.

Moreover, we found that neuroticism hampers happiness and reappraisal, and has a detrimental effect on suppression. Our results are consistent with those of previous studies [28, 33, 34]. One possible explanation is that neurotic people may exhibit maladaptive emotional coping (anger, sadness, and dissatisfaction), leading to anxiety and depression, which may negatively impact their enjoyment of life. Therefore, neurotic nursing students are susceptible to anxiety regarding their college requirements, which may hinder their enjoyment of life and happiness.

The current study found no significant differences between Jordan and Saudi Arabia in the associations between personality traits and happiness among nursing students. It is important to note that both Saudi Arabia and Jordan are Arab countries that share similar cultural and religious perspectives. In addition, nursing students in both countries study similar curricula. This result is supported by the finding that the nursing profession was initially started by Americans in both countries; therefore, it was based on the same American nursing guidelines and perspectives [35]. Additionally, both Saudi Arabian and Jordanian nursing students have been found to share the same perception of care provided to patients [36]. Additionally, there is a considerable proportion of Jordanian university faculty and nurses working in Saudi Arabia, which allows nursing students to share the same life and job perspectives and, therefore, a level of life satisfaction and happiness. Furthermore, nurses in Jordan and Saudi Arabia reported a moderate quality of nursing work life [37]. Therefore, it is not surprising that there were no differences in the relationship between personality traits and levels of happiness among nursing students in either country.

### Limitations

While this study reports significant results regarding personality traits and happiness among nursing students in Saudi Arabia and Jordan, it has some limitations. For instance, the study used a cross-sectional design based on self-reported questionnaires, which might hinder the establishment of causal relationships between the study variables and ignore the effect of time. Additionally, the use of online questionnaires may lead to bias in student responses. Furthermore, this study did not investigate other variables that might influence students’ levels of happiness and their relation to personality type in either country, such as academic load, academic achievement, extracurricular activities, level of stress, general health, and religious connection. Therefore, future studies should consider these limitations to explore the topic comprehensively.

## Conclusion

This is the first comparative study among nursing students in Jordan and Saudi Arabia that uses a structural equation modeling approach to investigate the relationships between personality traits, emotional regulation, and happiness. We found that while agreeableness and extraversion significantly enhanced happiness with a positive impact on reappraisal, neuroticism significantly impeded happiness and reappraisal. No differences in personality traits and happiness were found between the nursing students in Saudi Arabia and Jordan. Therefore, academic planners and decision makers are encouraged to plan programs that may enhance students’ personality trait identification and development to augment their happiness levels and emotional regulation.

### Recommendation

Future research should use longitudinal designs to establish causal relationships between personality traits, emotional regulation, and happiness among nursing students. It is crucial to consider variables such as academic workload, stress levels, and cultural nuances that are specific to Arab countries. It is recommended that intervention studies be conducted that focus on emotional regulation and employ qualitative methods to gain deeper insights. Further investigation is required to explore the impact of family relationships and leisure activities on happiness.

## Data Availability

The data that support the findings of this study are available on request from the corresponding author.

## Abbreviations

OHI: Oxford Happiness Inventory
ERQ: Emotion Regulation Questionnaire
SEM: Structural Equation Modeling
CR: Composite Reliability
AVE: Average Variance Extracted
VIF: Variance Inflation Factor
PLS-SEM: Partial Least Squares Structural Equation Modeling
MA: Multigroup Analysis
